# Cryo-EM structure of the bacteriophage T4 portal protein assembly at near-atomic resolution

**DOI:** 10.1038/ncomms8548

**Published:** 2015-07-06

**Authors:** Lei Sun, Xinzheng Zhang, Song Gao, Prashant A. Rao, Victor Padilla-Sanchez, Zhenguo Chen, Siyang Sun, Ye Xiang, Sriram Subramaniam, Venigalla B. Rao, Michael G. Rossmann

**Affiliations:** 1 Department of Biological Sciences, Purdue University, 240S. Martin Jischke Drive, West Lafayette, Indiana 47907-2032, USA; 2 Department of Biology, The Catholic University of America, 620 Michigan Ave. N.E., Washington, DC 20064, USA; 3 National Cancer Institute, National Institutes of Health, 50 South Drive, Bldg. 50 Room 4306, Bethesda, Maryland 20892, USA

## Abstract

The structure and assembly of bacteriophage T4 has been extensively studied. However, the detailed structure of the portal protein remained unknown. Here we report the structure of the bacteriophage T4 portal assembly, gene product 20 (gp20), determined by cryo-electron microscopy (cryo-EM) to 3.6 Å resolution. In addition, analysis of a 10 Å resolution cryo-EM map of an empty prolate T4 head shows how the dodecameric portal assembly interacts with the capsid protein gp23 at the special pentameric vertex. The gp20 structure also verifies that the portal assembly is required for initiating head assembly, for attachment of the packaging motor, and for participation in DNA packaging. Comparison of the *Myoviridae* T4 portal structure with the known portal structures of ϕ29, SPP1 and P22, representing *Podo*- and *Siphoviridae*, shows that the portal structure probably dates back to a time when self-replicating microorganisms were being established on Earth.

## Abstract (web summary)

Tailed bacteriophages translocate the genome into and out of the capsid through a portal protein assembly located between the phage s head and tail. Here Sun *et al*. provide a cryo-EM structure of the bacteriophage T4 portal protein assembly, suggesting the functions and evolution of the portal structure.

## Graphical abstract

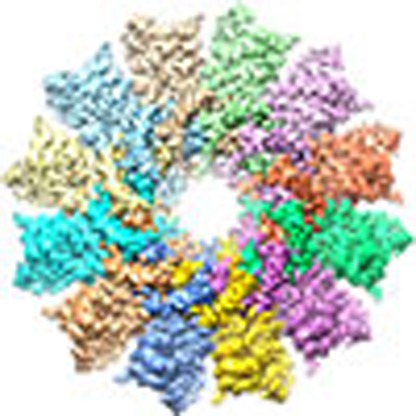

Tailed bacteriophages and herpesviruses translocate their genome into and out of their capsid (or ‘head’) through a 12-fold symmetric portal protein assembly (or simply ‘portal’), located at a special pentameric vertex of an icosahedral or prolate-icosahedral head^1^,^2^. The portal assembly serves at least three functions in the life cycle of most of the tailed phages^3^: (i) it initiates head assembly; (ii) it provides a platform for the packaging motor during DNA packaging into the prohead; and (iii) it is required for tail attachment. The degree to which the portal participates in DNA packaging or ejection is poorly understood.

The ‘small terminase’ of T4-like bacteriophages initiates DNA packaging into the prohead, a process that is powered by five copies of a virally coded ‘large terminase’ ATPase^2^,^3^,^4^. A variety of DNA packaging mechanisms had been proposed earlier, including the rotation of the portal assembly relative to the capsid^5^,^6^,^7^,^8^,^9^. However, the rotational hypothesis has now been ruled out based on mutational and structural data^10^,^11^. Most of the evidence suggests that the ATPase motor is both the power generator as well as the translocator of DNA^12^,^13^. However, the portal may not be merely acting as a passive channel for DNA transport. Mutational evidence suggests that it is also involved in DNA packaging^14^,^15^.

Bacteriophage T4 uses *Escherichia coli* as a host and belongs to the *Myoviridae* family of phages, characterized by contractile tails. Its structure and assembly have been extensively studied using biochemical, cryo-electron microscopic and X-ray crystallographic methods^16^. Many of the proteins that form the structure of the T4 capsid^17^,^18^,^19^,^20^, packaging motor^6^,^21^ and tail have been determined^22^,^23^,^24^. However, the detailed structure of the portal protein, gene product 20 (gp20), remained unknown^25^. Although the 61-kDa portal protein lacks recognizable transmembrane amino-acid sequences, unlike other portal proteins, it associates with the inner membrane of the host cell when initiating head assembly^26^. After the empty head has detached from the membrane, five large terminase proteins attach to the portal protein assembly^6^ creating the DNA packaging machine. When the packaging machine has completed filling the head with DNA, the gp17 molecules are jettisoned and the neck, tail and tail fibres are attached to complete the viral assembly^23^,^24^.

Portal proteins from different phages have different molecular masses. Furthermore, there is no detectable sequence similarity between the portal proteins from different types of phages. Nevertheless, crystal structures of the ϕ29, SPP1 and P22 phage portal protein assemblies show considerable similarity^5^,^27^,^28^. The monomers can be divided into separate clip, stem, wing and crown domains. Although the overall structures of the dodecamers vary, all of them have a conserved central clip-stem-wing topology. The crown domain is disordered in the ϕ29 portal assembly^5^. The portal protein assemblies have funnel-like external shapes with a length of about 75 Å for ϕ29 and about 110 Å for the SPP1 and P22 portal structures. The wide end of the funnel is inside the capsid. Their central channel has a minimum diameter of about 28 Å. The portal protein assembly of phage P22 has an additional 200 Å-long carboxy-terminal, α-helical, coiled coil domain inside the head that might facilitate genome spooling onto the interior surface of the capsid during genome packaging and also facilitate genome ejection into the host cell during infection^28^,^29^.

In this paper we describe the cryo-EM structures of gp20 portal protein at 3.6 Å resolution and the empty prolate T4 head at 10 Å resolution. The function of the portal assemblies in bacterial phages is clarified by comparing the T4 portal structure with the known portal structures of *Siphoviridae* and *Podoviridae* and by combining the structural information with mutational data.

## Results

### Cryo-EM structure determination of the T4 portal assembly

The structure of the T4 portal protein assembly was studied using both X-ray crystallography and cryo-EM. Although the best crystals of the portal protein diffracted to only 6.5 Å resolution, we have been able to produce a single particle cryo-EM reconstruction that has about 3.6 Å resolution. We attribute the greater success with cryo-EM as compared with X-ray crystallography to the computational selection of particles with good homogenous quality in the EM studies than is possible by crystallization. The molecular weight of the T4 portal assembly construct used for this study is 660 kDa, somewhat small for achieving a good cryo-EM reconstruction. Nevertheless, with the help of a direct electron counting detector, it was possible to obtain a 3.6 Å resolution electron density map.

The 524 residue phage T4 portal protein is extremely insoluble. To improve the solubility, numerous constructs and different purification protocols were screened (Supplementary Table 1). Of all these attempts, the construct gp20-N74 (residues 74–524) was found to have the best solubility and to give the best crystals. However, even these crystals diffracted X-rays to only 6.5 Å resolution. Therefore, an attempt was made to determine the portal structure using cryo-EM. The initial cryo-EM data of N74 showed that about 90% of the particles were 12-mers, with the rest being 11-mers (0.1%) or 13-mers (9.9%) (Supplementary Fig. 1c). Even to obtain this level of homogeneity it was found necessary to collect a very narrow gel-filtration peak (500 μl out of a 12 ml wide peak). Different oligomeric states in this sample may have been the main factor that limited the X-ray diffraction resolution of the crystals. A second gel-filtration purification was necessary to obtain the highest purity of 12-mers (95%), although there still was a small percentage of 13-mers (5%).

**Figure 1:**
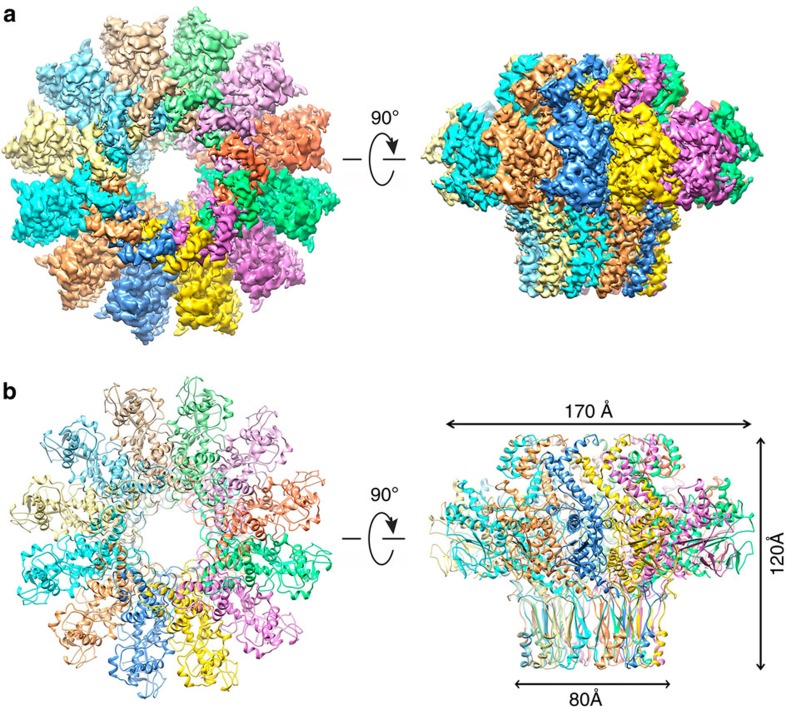
Cryo-EM structure of the T4 portal protein (gp20) assembly. (**a**) 3D density map of T4 portal protein assembly at 3.6 Å resolution with each subunit colour-coded. Shown are the top view (left) and side view (right). (**b**) Ribbon diagram of the gp20 atomic model with each subunit colour-coded. Shown are the top view (left) and side view (right).

The gp20-N74 protein was used to collect EM data with an FEI Titan Krios microscope equipped with a Gatan K2 Summit direct electron detector. A total of about 98,000 particles were boxed from 839 selected averaged images (see Methods). Aberrant particles could be recognized and then discarded in the two-dimensional classification procedure. The reconstruction assumed 12-fold symmetry. An early difficulty in determining the structure was the preference by the particles to orient themselves with their 12-fold axes perpendicular to the ice surface, resulting in a dearth of side views. This was partially rectified by adding octyl-β-glucoside to the sample before freezing (Supplementary Fig. 1a,b). The final three-dimensional cryo-EM reconstruction had an overall resolution of 3.6 Å (Fig. 1a), based on the ‘gold-standard’ Fourier shell correlation (FSC) using the FSC=0.143 criterion (Supplementary Fig. 1e). The map showed continuous density for the polypeptide chains and recognizable side-chain densities (Supplementary Fig. 2). An atomic model was built based on this map, using the COOT graphical program^30^. The crystallographic program Phenix^31^ were used to refine the model (Fig. 1b) by minimizing the vector difference between the Fourier coefficients obtained by back-transforming the final cryo-EM map and the calculated structure factors derived from the atomic model. The final *R* (working) and *R* (free) values were 0.26 and 0.27, respectively (Supplementary Table 2).

### Structure of the portal assembly

**Figure 2:**
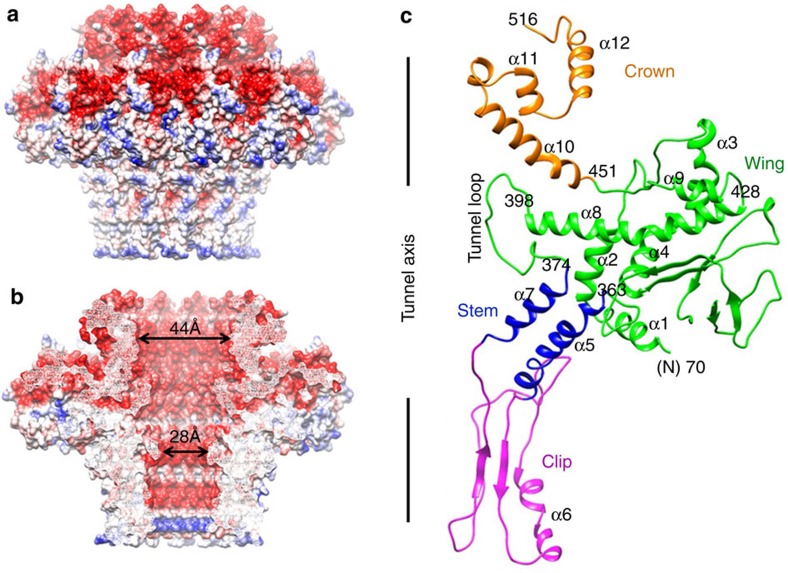
The structure of gp20. (**a**) Charge distribution on the outer surface of dodecameric gp20. Blue and red colours correspond to 10 kT e^−^ positive and negative potential, respectively. (**b**) Charge distribution on the inner surface of dodecameric gp20. (**c**) Ribbon drawing of the gp20 monomer structure with each domain colour-coded.

The T4 portal protein assembly is dodecameric and has a length of ∼120 Å (Fig. 1). The external diameter of the portal complex varies from 90 Å at the ‘upper’ end to 170 Å in the middle and to 80 Å at the ‘lower’ end close to the outside the capsid. The central channel has a diameter of about 44 Å at the upper end, decreasing to 28 Å at the narrowest point near the middle of the cylinder (Fig. 2b). The crown domain (residues 451–524) consists of three α-helices (α10, α11 and α12) connected by short turns and eight additional disordered C-terminal residues (Fig. 2c). The wing domain (residues 70–254 and 378–450) consists of α-helices α1, α2, α3, α4, α8 and α9 and forms the central part of the portal assembly. A bent helix, α8, is 30-residues long (residues 399–428) and is connected to the stem region (residues 255–278 and 359–377) by the ‘tunnel’ loop (residues 374–398). This loop protrudes into the central channel of the portal assembly and has lower density than the rest of the structure, suggesting flexibility. In the portal structures of the SPP1, P22 and ϕ29 this loop is 14, 10 and 23 amino-acid long, respectively, and is mostly completely disordered. The stem region consists of two helices, α5 and α7, that are oriented at an angle of about 40° with respect to the central 12-fold axis and are connected to each other by the clip domain. The clip domain (residues 279–358) consists of three β-strands and helix α6. This domain is exposed on the outside of the capsid. It is the site of attachment of the packaging ATPase, gp17, during DNA packaging, as well as for attachment of the neck (gp13) and tail in the mature phage.

The contact area between adjacent subunits of the T4 portal assembly is 4,100 Å^2^. This contact is, in part, stabilized by the clip region that contains a three-stranded β-sheet, consisting of one β-strand from one subunit and two β-strands from an adjacent subunit (Supplementary Fig. 3a). The hydrogen bonds in this interaction presumably provide a significant component of energy for the portal protein assembly. This is the only region with direct main-chain/main-chain inter-subunit hydrogen bonds. The rest of the subunit interface is stabilized mainly by seven salt bridges between the adjacent neighbouring surfaces (Supplementary Fig. 3a). A similar interface has been observed in the ϕ29, SPP1 and P22 portals^5^,^27^,^28^. The high ionic interaction in the T4 portal may explain the observation that EDTA and high salt concentration are necessary to keep the T4 portal assembly stable in buffer.

### The interaction of the portal with the capsid

**Figure 3:**
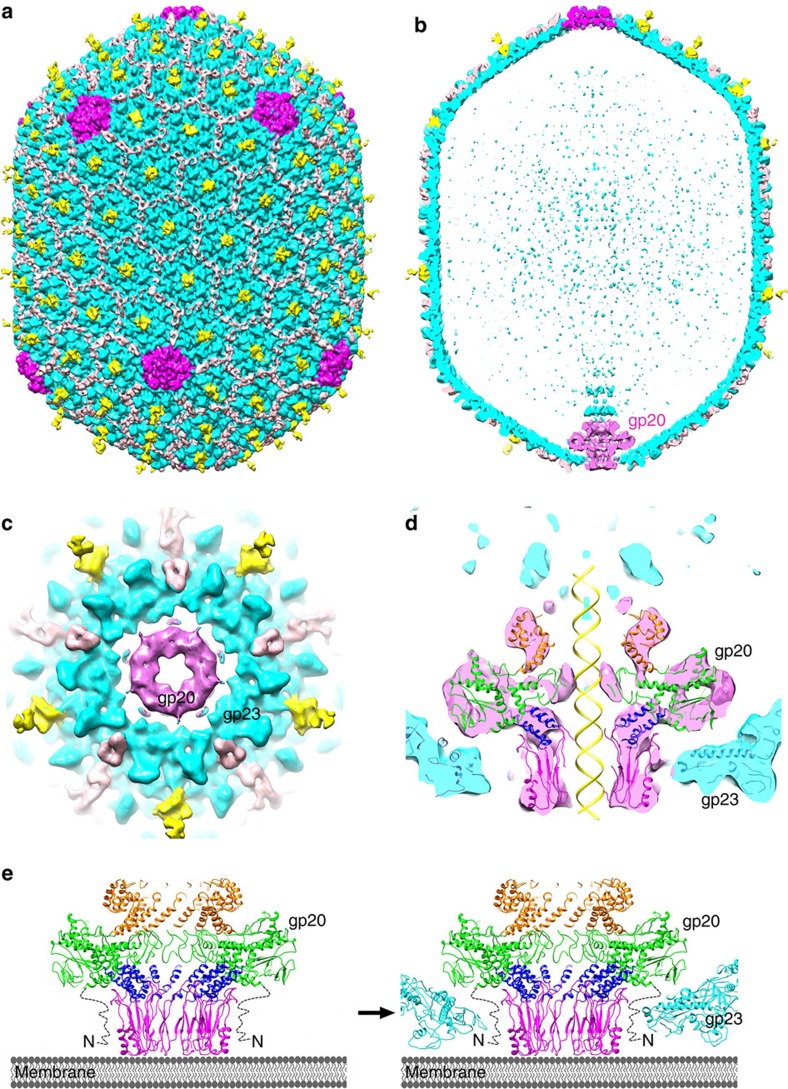
Cryo-EM reconstruction of the T4 prolate head at 10 Å resolution. (**a**,**b**) Cryo-EM density map of the T4 prolate head (gp23: cyan; gp24:magenta; Soc: pink; Hoc: yellow). (**c**) Bottom view of the prolate head, showing the gap between gp20 and the capsid. (**d**) Fit of the gp20 and gp23 structures into the cryo-EM map of the T4 prolate head. (**e**) A model of the T4 head assembly. A dodecameric portal is assembled on the inner membrane of *E. coli* with the assistance of the phage-coded chaperone gp40 and the *E. coli* chaperone YidC^58^. The portal assembly acts as an initiator for head assembly, leading to co-polymerization of the major capsid protein gp23 and scaffolding proteins.

A three-dimensional, cryo-EM structure of the mature T4 prolate head-plus-tail had been determined to 22 Å resolution using fivefold symmetry^17^. In addition, the structure of the prolate prohead with the attached packaging motor had been determined to 35 Å resolution using fivefold symmetry^6^. Here we also report a 10 Å resolution reconstruction of an emptied prolate head, determined by imposing fivefold symmetry. All these reconstructions show the small outer capsid protein (Soc) molecules surrounding the major capsid protein (gp23) hexamers and the highly antigenic outer capsid protein (Hoc) molecules protruding from the centre of the hexamers^18^,^20^ (Fig. 3a). Furthermore the fivefold vertices are occupied by gp24 pentamers. The special vertex is missing this pentameric unit and instead contains the portal assembly. Because of the 12-fold to 5-fold symmetry mismatch between the portal and capsid, respectively, there remains uncertainty about the relative rotational orientation of the portal with respect to the capsid. Thus, the cryo-EM density representing the portal in the prolate head is the average of many orientations and, therefore, represents the envelope of the portal assembly. A model of the major capsid protein, gp23, derived from a 5.6 Å resolution reconstruction of an isometric T4 mutant (Z. Chen, Z. Zhang, L. Sun, M. G. Rossmann, V. B. Rao, manuscript in preparation) was used to interpret the structure of the prolate head.

Given the above knowledge of the capsid, the T4 portal structure, based on the 3.6 Å resolution cryo-EM map described above, was fitted into the 10 Å resolution structure of the prolate head. The gp20 portal was found to be located in a wide tube, or hole, at the special vertex of the capsid (Fig. 3b). This aperture, created by the absence of the gp24 pentamer, is about 25 Å long and 110 Å in diameter. There is a gap, roughly 15 Å in width, between the external face of the portal assembly and the internal face of the 5-fold symmetric tube/hole at the special vertex (Fig. 3c). Thus, the contact between the portal and the capsid is primarily through the portal proteins’ wing domains resulting in about 819 Å^2^ contact area per portal monomer. The interaction between the wing domains and the capsid are partially hydrophobic and partially polar (Supplementary Table 3). This would make it difficult for the portal to rotate relative to the capsid during DNA entry or exit^10^ because a rotation would result in the polar residues on gp20 coming in contact with hydrophobic residues on gp23.

**Figure 4:**
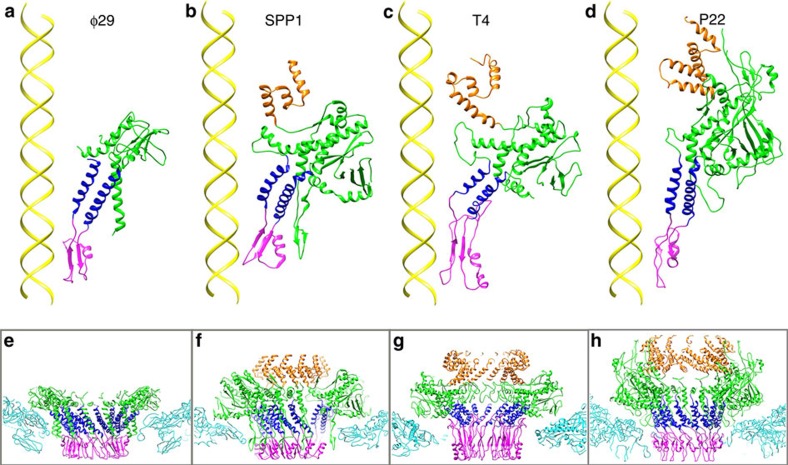
Comparison of the four known portal proteins. The upper row (**a**–**d**) shows the different portal protein subunits with their wing, stem, clip and crown domains coloured green, blue, purple and orange, respectively (PDB IDs of portals: ϕ29: 1FOU, SPP1: 2JES, P22: 3LJ4). The lower row (**e**–**h**) shows the portal assemblies docked into their respective phage capsids (cyan). The T4 portal structure was fitted into the 10 Å resolution EM map of the prolate head. Similarly the other portal structures were docked into their capsid structures (PDB IDs: ϕ29: 1YXN, P22: 2XYZ, SPP1: 4AN5).

The T4 portal model is missing the first 73 residues. However, there is no extra density in the 10 Å resolution prolate prohead cryo-EM reconstruction that might account for these missing residues, suggesting that these residues are disordered. The only available space for these residues appears to be the ‘empty’ region between the external surface of the portal and the internal surface of the tube or the hole formed by the capsid, created by the absence of the gp24 vertex protein (Fig. 3c,d). Therefore, it is perhaps reasonable to place these residues snuggly around the outer surface of the portal, as a part of the wing domain, similar to the other portal structures (Fig. 3e and Fig. 4). In addition, this would leave the wing domains accessible to bind gp23 hexamers, initiating further hexamer binding until the complete head has been formed (Fig. 3e), consistent with the expectation that the portal is required to initiate head assembly^3^. Indeed, mutational analysis shows that deletion of the N-terminal region inhibits the head assembly (Supplementary Table 1).

### The interface of the portal with the DNA packaging motor

**Figure 5:**
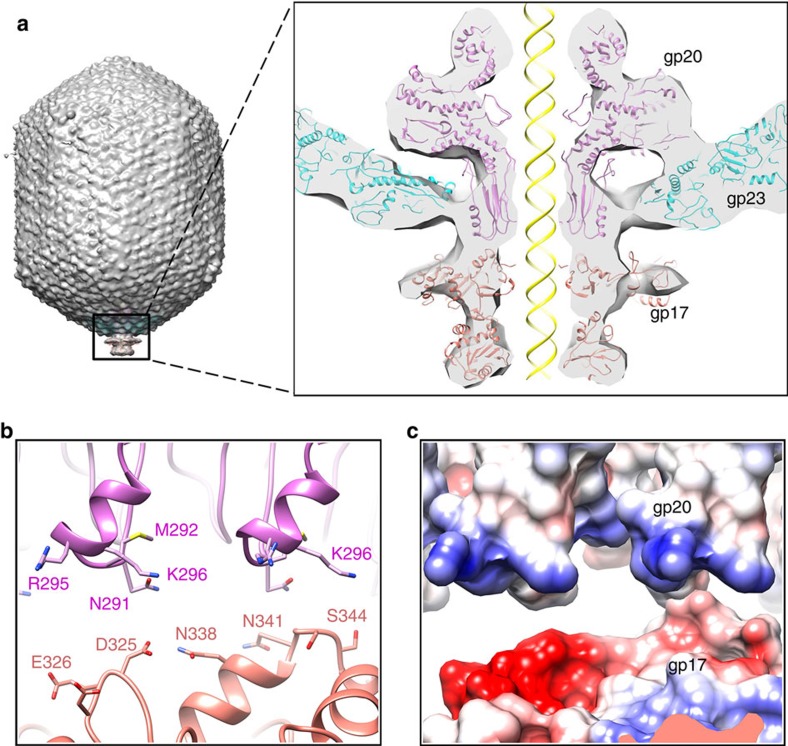
Interactions between the T4 gp20 portal and the gp17 ATPase. (**a**) Fitting of the T4 portal protein (purple) and gp17 (tan) into the 35 Å cryo-EM reconstruction of the procapsid+gp17 (EMD-1572 accession number). (**b**) Residues involved in the interaction between gp20 (purple) and gp17 (tan) are shown as sticks. (**c**) The surface charge of gp20 and gp17 around the interface area showing electrostatic interactions. The view orientation is the same as in panel (**b**).

The structures of the T4 gp20 portal protein assembly and of the T4 large terminase gp17 ATPase were docked into the previously determined 35 Å resolution cryo-EM structure of the prolate prohead complexed with gp17 (ref. 6). The docked structure shows that the five gp17 ATPases bind to the portal protein assembly with no apparent contact between the gp17 molecules and the capsid protein (Fig. 5a). The area of contact between the ring of twelve gp20 molecules and the five gp17 molecules involves primarily electrostatic interactions.

The residues at the tip of the portal protein’s clip domain (N291, M292, R295, K296) protrude out of the capsid and probably interact with the gp17 ATPase residues D325, E326, N338, N341 S344, forming strong charge interactions (Fig. 5b,c). Mutating residues of gp17 in this interface resulted in a loss of DNA packaging activity (for example, GSS342–344 to AAA) or in a heat-sensitive phenotype (for example, DD330–331 to DA or CD)^32^. Similarly, mutation of residues in this region of the portal protein (for example, N291A-M292A, R295A-K296A) abolished DNA packaging^14^, presumably because of interference in the way gp17 binds to the prohead (Fig. 5b). An M292I mutation resulted in cold-sensitive phage, which under restrictive conditions failed to initiate DNA packaging and accumulated empty proheads^33^,^34^. Consistent with these observations, a 24-amino-acid peptide corresponding to the residues 288–311 can bind to gp17 and inhibit DNA packaging^34^.

All suggested mechanisms for DNA packaging into the capsid of T4^6^,^9^,^14^ or into the capsid of ϕ29 (refs 5, 35, 36) assume that the five different ATPases take turns to hydrolyse ATP in a defined order. Because B-DNA has a 10-fold screw axis and the portal protein has 12-fold symmetry, the contact of each gp20 monomer with DNA will be different. This asymmetric contact of the DNA with the portal assembly suggests that there is a unique information pathway from one gp17 ATPase to the next ATPase that determines the order in which each ATPase becomes activated.

### Interaction of the T4 portal with DNA

**Figure 6:**
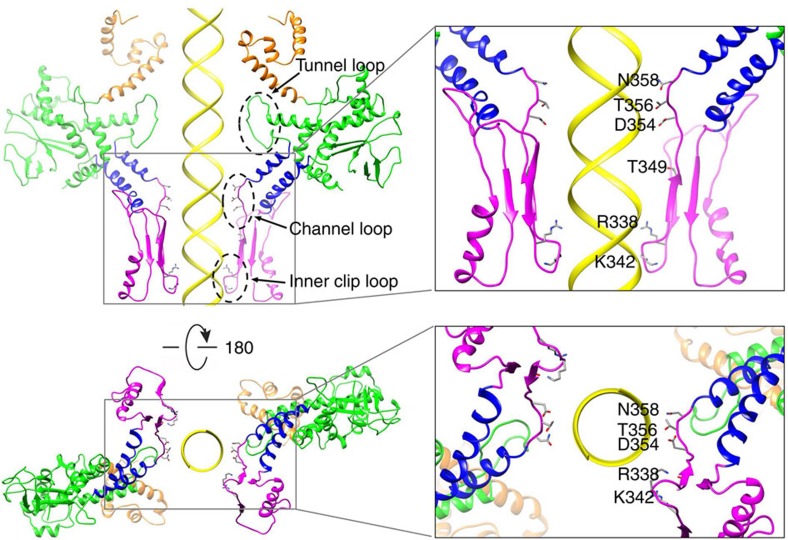
The gp20 structure with modelled dsDNA showing the key residues on the three loops (dashed black circles) that interact with DNA. The different portal protein subunits with their wing, stem, clip and crown domains are coloured green, blue, purple and orange, respectively.

Fitting of a B-DNA structure into the central channel of the dodecameric portal assembly shows that the contacts between any one gp20 molecule and the DNA are confined to three polypeptide loops separated from each other by approximately one DNA helical turn. These occur at the end of the clip domain (inner clip loop), at the loop near the amino end of α7 in the stem domain (channel loop) and at the tunnel loop (Fig. 6).

The inner clip loop contains two basic residues (R338 and K342) that are present in each of the 12 portal protein monomers, pointing into the DNA channel (Fig. 6). These 24 residues surround the entrance of the portal channel, suggesting that these positive charges might be important to capture the start of the viral genome to initiate DNA packaging. Mutational studies showed that changing these basic residues to alanine resulted in loss of DNA packaging^14^. Nevertheless, the positive charges at the entrance of the T4 portal channel are not conserved in other phage portals, leaving uncertain the function of the positive charges at the entrance to the channel.

The channel loop (residues 350–359) connects the clip domain to the stem helix α7. It forms one of the constrictions of the portal channel reducing the diameter to ∼28 Å (Fig. 2). This loop might interact with the DNA during translocation into or out of the capsid. This region is exceptionally well conserved in the amino-acid sequence between T4 and SPP1 (Supplementary Fig. 4). In the SPP1 phage portal protein the T319A mutation, corresponding to T349 in gp20 (Fig. 6), resulted in poor stimulation of the ATPase and DNA packaging activities. Possibly amino-acid changes in the loop might cause interference in the DNA translocation that, in turn, might impact the sequence in which the gp17 ATPases hydrolyse their substrate.

The tunnel loop consists of 26 residues (amino acids 374–398) between helix α7 and the bent helix α8. The tunnel loop protrudes into the channel and has only weak density in the isolated portal. However, in the emptied head it is well ordered and forms the narrowest part of the channel, too narrow to allow the passage of DNA (Fig. 3d). Thus, the conformation of the loop is variable and probably controls the passage of DNA when disordered (‘open’) or stops the packaged DNA from coming back out (‘closed’). Furthermore, cryo-EM micrographs of a mutant of gp20 (Supplementary Table 1) in which the tunnel loop (residues 377–388) had been deleted showed that about 28% of the purified mutant portals were 11-mers and 4% were 13-mers (Supplementary Fig. 1d), compared with 0.1 and 9.9% in gp20-N74, respectively, when the tunnel loop was present (Supplementary Fig. 1c), implying that the tunnel-loop may help to assemble the dodecamers. This is probably because the tunnel loops interact with the tunnel loops of the neighbouring subunits (Supplementary Fig. 3b). The tunnel loop-deletion mutant did not affect packaging of short DNAs but was unable to complete the packaging of the full-length genome resulting in non-infectious virions^14^. A similar mutant in the ϕ29 tunnel loop could not complete packaging of the full-length genome^37^. Possibly the tunnel loop prevents the DNA from slipping back out of the head. This might be more critical in the late stage of DNA filling where repeated attempts at completing packaging fail because the internal pressure pushes the DNA back out after every cycle of packaging.

### Comparison with other portal proteins

The structures of the ϕ29, SPP1, P22 and the T4 portal assembly are now all known (Fig. 4 and Supplementary Table 4). Their monomeric structures were superimposed pairwise using the program HOMOLOGY^38^ to establish the sequence alignment of the structurally equivalenced Cα atoms (Supplementary Fig. 4). On average, the residues that had the smallest separation between structurally equivalenced Cα atoms were found to have the greatest chemical similarity as measured by the average minimum base change per codon. Similar observations have been made for other conserved folds^39^. Conservation of these residues is probably required to guide the folding process to maintain topologically similar structures during divergent evolution of these proteins.

The structurally aligned sequences show four sites at which three of the four portal structures have the same residues (Supplementary Fig. 4). Two of these sites are spatially close together and conserve a hydrophobic centre of the portal subunit. Sequence identity of the structurally aligned residues between any two portal proteins does not exceed 12% (Supplementary Table 5) suggesting that, if they deviated from a common origin, the ancient portal protein is likely to have arisen at a time close to the origin of tailed bacteriophages. The percentage of residues that can be structurally equivalenced relative to the total length of the proteins is mostly <50% (Supplementary Table 6). This is less than the percentage of residues that are structurally conserved when comparing nucleotide binding structures that are likely to have been present during pre-cellular evolution^40^. This suggests that ‘viruses’ with portal assemblies could have occurred even before life was fully established on Earth.

When there is little evidence for a common origin in amino-acid sequences, the evolutionary separation between any two homologous structures can be measured in terms of structural similarity as, for instance, by the number of Cα atoms (*r*) (Supplementary Table 7) that can be superimposed between pairs of structures. This number defines the size of the common fragment between any pair of structures. There is an inverse relationship between the number of residues that can be structurally equivalenced and the time that has occurred since the separation from a common origin. Thus, the evolutionary distance could be roughly proportional to either (*P*—*r*) or (100*P*/*r*), where P is a constant that is roughly the number of residues that constitute a functioning portal protein (Supplementary Table 7). The crown domain was omitted from these measurements because it is in a different orientation in each structure.

**Figure 7:**
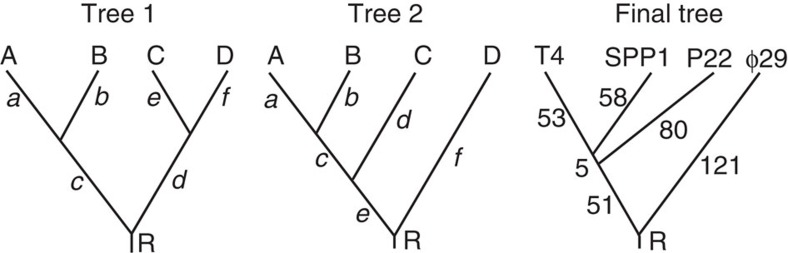
Left and centre: possible rooted phylogenetic trees (tree 1 and tree 2) that might represent the evolution of the primordial portal protein *R* to the current portal proteins of bacteriophages (**a**–**d**). The lengths of the branches are labelled as (**a**,**b**,**d**–**f**). The tree on the right shows the tree that best fitted the structural observations.

There are two types of rooted, phylogenetic trees (‘tree 1’ and ‘tree 2’) that can be drawn for the evolution of four known structures each with seven parameters defining the lengths of the branches of the trees (Fig. 7). There are 12 different ways of arranging the four structures at the end of the branches furthest from the root. However, tree 1 has symmetry allowing for only three unique tree varieties. Hence, in total, there are three varieties of tree 1 and twelve varieties of tree 2.

The process of constructing phylogenetic trees that are likely to represent evolutionary events has been a subject of extensive discussion and development of software packages^41^,^42^. In the present case six linear observational equations can be written defining the distance between the portal structures and four further observational equations can be written defining the distance of each structure from the root of the tree (see Methods). This gives a total of 10 linear observational equations to determine seven parameters. These were determined by a linear least squares procedure for each of the possible 15 tree varieties (Supplementary Table 7). The results were similar whether the evolutionary distance was calculated by the (*P*−*r*) or (100*P*/*r*) definition of evolutionary distance. The resultant best (lowest *R* factor) and physically meaningful (no negative branch lengths) tree was tree 2.1 (Supplementary Table 7). This tree shows the portal protein of T4 to be related most closely to the portal protein of SPP1. It also shows ϕ29 to be the most distantly related to the other portal proteins, consistent with the observation that ϕ29, unlike the other portal proteins, is missing most of the crown domain, suggesting that the crown domain performs another function that is not required by ϕ29. The crown domain is the most internal component of the T4, SPP1 and P22 portal proteins and extends the channel of the portal towards the centre of the virus. It may, therefore, be relevant that P22 has an additional helical barrel domain extension that channels the DNA into or out of the head and T4 also has an additional internal feature observable in the cryo-EM reconstruction of the prolate head (Fig. 3b). The internal channel extension of the portal in T4 might possibly be created by cleaved scaffolding proteins that remain inside the head (Internal Proteins IP I-III, gpAlt)^43^.

## Discussion

All the portal assemblies show a conserved protein fold, despite the less than 12% sequence identity based on structural comparisons. It has long been established that, in general, the fold of a protein changes more slowly than amino-acid sequences^40^. Furthermore, changes in the fold of a protein are generally constrained by conservation of function^38^,^39^,^40^,^44^. Thus, the conservation of fold of the portal assemblies in *Myoviridae* (T4), *Siphoviridae* (SPP1) and *Podoviridae* (ϕ29 and P22) tailed phages attests to the functional importance of the portal assembly to the life cycle of tailed phages.

The principal differences between the four portal structures are primarily in the hinge angle between domains within the portal proteins and their relationship to the common 12-fold axis (Fig. 4, Supplementary Fig. 5 and Supplementary Table 8). The portal assembly provides the interface between the capsid and DNA during DNA packaging or ejection. The portal assembly must, therefore, adapt itself on the one hand to the different structural properties of the various phage capsids and on the other to the different requirements during DNA packaging and ejection. As different phages have different head structures the interaction of the portal with the capsid results in different angles of the portal domains relative to the central 12-fold rotation axis (Fig. 4 and Supplementary Table 8). In contrast, the DNA structure always makes similar contact with the tunnel loop and the clip domain in all four portal proteins (Fig. 4).

Adaptation of the portal proteins to their respective capsid structures is manifested by the angles between the wing domain and the stem domain with the internal surface of the various capsid structures^45^,^46^,^47^ (Fig. 4). Furthermore, the clip domain straddles the thickness of the capsid walls by matching its structure with the thinner capsid walls in SPP1 and the thicker capsid walls in the other known portal structures. Adaptation of the portal structure to the requirement of DNA might be dependent on the interaction of the clip, stem and wing domains with the DNA in the portal channel. These interactions are, in part, created by the preponderance of negative charge on the inner surface of the portal assembly to permit the smooth passage of DNA during packaging and ejection (Supplementary Fig. 6). The presence of two lysine rings in the channel surface of ϕ29 might contribute to its lower packaging rate (∼100 bp s^−1^) compared with the T4 packaging rate (∼800 bp s^−1^)^48^,^49^. In addition, the ‘upper’ external surfaces of the portal structures are also highly negatively charged, presumably to avoid binding of the packaged DNA to the portal (Fig. 2a and Supplementary Fig. 6).

The structures of the four known portal structures were determined from different crystals or, in the case of T4, from no crystal at all. Thus, the lattice forces acting on the portal would be unique for each of the known structures. The different domains in these structures make different angles relative to each other (Supplementary Table 8). Thus, either these portals are each rigid but different, or they are flexible and are ‘frozen’ into a specific orientation by the lattice forces acting on them. If the latter were the case, then the different portal structures that are now known might represent intermediates that occur during viral maturation, DNA packaging, or ejection.

The T4 portal assembly might also act as a pressure gauge by being in contact with the DNA at three different loops and in contact with the packaging motor to regulate motor function. This is consistent with mutations in the tunnel or channel loops of SPP1 and P22 portals causing too much or too little DNA being packaged into the head^15^,^50^. Furthermore, the portal might function to close the twelve tunnel loops to stop the DNA from coming back out once the head has been filled^14^,^37^.

With the availability of four portal structures from *Myo*-, *Podo*- and *Siphoviridae* tailed phages it was possible to make a study of the evolution of these phages from a primordial phage. Without the structural information it would not have been possible to detect the small amount of amino-acid sequence similarity, indicating that the divergence from a common origin is rather ancient.

## Methods

### Cloning of gp20

DNA fragments containing gene (g) 20, g13 or g22 were amplified by the polymerase chain reaction (PCR) using Phusion (New England Biolabs) or KOD DNA polymerase (NOVA, LCP Biomed, Lianyungang, China) with appropriate primers. Specific primers were used to amplify the desired DNA sequences (Supplementary Table 1). Point mutations and tunnel loop deletions were introduced using mutant primers with the ‘splicing by overlap extension’ strategy described previously^51^,^52^. The amplified DNA fragments were digested with appropriate restriction enzymes (Thermo Scientific, Waltham, MA) and ligated into the pET-28b (+) or pCDFDuet-1 vector. The ligated DNAs were transformed into *E. coli* XL10-Gold Ultracompetent Cells (Agilent Technologies, Santa Clara, CA) for a miniprep plasmid DNA preparation. The accuracy of the cloned DNA was determined by DNA sequencing (Retrogen, San Diego, CA). Where necessary, the 5′-end primers were designed such that ligation of the spliced DNA into the vector will result in in-frame fusion with a hexa-histidine tag (His-tag) sequence in the vector. For the gp20-N74 (residues 74–524) and gp22 co-expression constructs, the g20 DNA fragments were inserted into the multiple cloning site 1 of pCDFDuet-1 vector to fuse the His-tag at the N-terminus (gp20 primers: F: 5′- CGCGGATCC GGAGCTTATTGATACATATCGT -3′; R: 5′- ATAAGAATGCGGCCGCTTAAAAATCCTCTTG TTCTTG -3′). In the second step the g22 DNA was inserted into the multiple cloning site 2 of the recombinant plasmid (gp22 primers: F: 5′- CGCGCGATATCGATGCTTAAAGAACAACTGA TT -3′; R: 5′- CCGCTCGAGTTAGAAACGAGATGCGACTTT -3′), resulting in the co-expression of His-tagged gp20 and non-tagged gp22.

### Expression and purification of the gp20-N74

The cryo-EM structure was determined using the clone that co-expresses gp20 (no tag) and gp22 (with an N-His tag) (Supplementary Table 1). For expression of the gp20 protein, the plasmid DNA was transformed into *E. coli* BL21(DE3) pLysS-competent cells (Novagen) and induced with IPTG at 20 °C for 16 h. For purification of gp20, the cells were harvested by centrifugation at 6,000 r.p.m. for 15 min at 4 °C. The bacterial cell pellet was resuspended in 20 mM Tris-HCl (pH 7.8), 400 mM NaCl, 1 M Urea, 20 mM imidazole, 5% glycerol (w/v) and homogenized by sonication. The cell lysate was centrifuged at 16,000 r.p.m. for 30 min at 4 °C to remove cellular debris. The supernatant fraction was loaded onto a HisTrap 5 ml column (GE Healthcare). The protein complexes (gp20-gp22) were eluted with a buffer containing 20 mM Tris-HCl (pH 7.8), 400 mM NaCl, 1 M Urea, 20 mM imidazole and 5% glycerol (w/v). The protein complexes were further purified using size-exclusion column Sephacryl 16/60 HR S-400 (GE Healthcare) in a buffer containing 20 mM Tris-HCl (pH 7.8), 400 mM NaCl and 5 mM EDTA. High salt concentration was found necessary to prevent protein precipitation. EDTA was used to prevent protein from aggregation. Gp20 and gp22 were eluted from the size-exclusion column as two separate peaks. A small fraction (500 μl) of the gp20 peak was collected and further purified by using a Superose 6 (GE Healthcare) size-exclusion column. The protein was concentrated to 1 mg ml^−1^ for cryo-EM experiments. An attempt at further purification using a MonoQ ion exchange column failed to separate other oligomers (11-mers and 13-mers) from the major fraction of 12-mers.

### Preparation of gp20 for cryo-EM

Fresh gp20 protein was buffered with 20 mM Tris-HCl (pH 7.8), 400 mM NaCl and 5 mM EDTA with a concentration of 1 mg ml^−1^. To avoid protein orientation preference on the frozen grids, 0.3% of octyl-β-glucoside was added to the sample just before sample freezing. The gp20 sample was frozen onto quantifoil grids using a cryo plunge chamber (CP3) with a blotting time of 7 s while the humidity was maintained above 85%.

### Data collection and processing of gp20

The frozen EM grids with the gp20 protein were first screened using the Purdue University Titan Krios operated at a voltage of 80 kV (to increase contrast) and equipped with a charge-coupled device (CCD) camera. Grids with good protein concentration and protein orientation distribution were saved after they were unloaded from the Titan Krios for further data collection. The gp20 data were collected using a Gatan K2 summit direct detector and while using the counting mode on the Titan Krios at the University of California, Los Angeles (UCLA), operated at 300 kV. Automatic data collection using the Leginon program^53^ was controlled remotely at Purdue University. The automatic data collection was finished within 24 h and produced 866 images. Each image was composed of 24 frames, each with an exposure time of 0.25 s and a dose rate of about 2 electrons per Å^2^.

Averaged images were produced from the 24 frames after motion correction^54^. These were used for boxing the particles. The program e2boxer.py^55^ was used to automatically box individual particles. The program ctffind3^56^ was used to determine the parameters for the contrast transfer function (CTF). A total of about 98,000 particles were boxed from 839 selected averaged images. A reference-free, two-dimensional (2D) classification program implanted in the RELION software^57^ was used to classify the boxed particles by a reciprocal space maximum likelihood method. After 2D classification, 79,868 particles within 15 selected classes from a total of 150 classes were selected for further data processing. The class representing the top view of the protein showed that gp20 formed dodecamers. To create an initial model for defining classes, an electron density was calculated to 100 Å resolution based on the atomic structure of the SPP1 13mer. This density was then averaged by imposing 12-fold symmetry. To refine the three-dimensional (3D) model, the same particles were re-boxed from the averaged images using only frames 2 to 12 while excluding the frames with large movements and greater than average radiation damage^54^. The program RELION was used for 3D auto-refinement^57^. The data set of re-boxed particles was split into two halves before the start of the refinement. The low resolution model was then refined in each half data set independently. The refinement stopped when there was no further improvement of the Fourier shell correlation coefficient (FSC). The FSC was calculated from the two independent reconstructions. The resolution of the reconstruction was determined by the point at which the FSC=0.143. A temperature factor of −100 Å^2^ and a low pass filter to 3.6 Å were applied to the whole data set for calculating the final map.

### Atomic model building and refinement of gp20

The map shows continuous density for the main chain and distinct side-chain densities for bulky amino-acid residues such as arginine, tryptophan, phenylalanine and tyrosine. Taking advantage of regular bumps representing the Cα atoms, a model of the backbone was built from scratch using the program COOT^30^. Amino-acid registration was accomplished based on the clear densities for ‘landmark’ stretches of residues. The full-atom model was refined against the structure factors obtained by Fourier back-transforming the cryo-EM map using the Phenix program^31^ assuming a triclinic cell with 12-fold non-crystallographic symmetry (Supplementary Table 2). This procedure was used to improve the fit of the atomic model to the EM density map. Further improvement was achieved by iterative cycles of manual model rebuilding and pseudo-crystallographic refinement. The quality of the model was verified by visual inspection and by calculating the crystallographic working and free R factors (26%/27%). Out of the 459 amino-acid residues per monomer (including eight residues from the plasmid vector into which the gp20 gene had been inserted), 436 amino acids could be located in the electron density map. The missing residues were 192 to 203 and 517 to 524, all located on the outer surface of the portal.

### Cryo-EM study of T4 emptied prolate head

The neck proteins gp13, gp14 and gp15 assemble onto the portal of the packaged head before tail attachment. A gp13 amber mutant produces DNA-full heads. During purification, about 90% of these mutant heads release most of the packaged DNA, leaving an ∼8 kb piece of DNA in each head. The heads are fully expanded and have a prolate shape. They are decorated with Hoc and Soc proteins, which increase their stability and homogeneity. The purified heads were suspended in a buffer containing 50 mM Tris pH 7.5, 1 mM MgCl_2_ and 100 mM NaCl. Grids were frozen in liquid ethane using the CP3 plunger. Cryo-EM film data were collected using an FEI Titan Krios electron microscope with a 59,000 magnification at Purdue University. Approximately 13,000 particles were boxed. A 52-symmetry reconstruction was calculated to about 9 Å resolution. The symmetry was then relaxed to only fivefold, resulting in a 10 Å resolution reconstruction.

### Determining the most likely phylogenetic tree

The portal protein structures were compared pairwise using the HOMOLOGY program^38^. The results were used to produce manually a table giving the sequence alignment of all four known portal structures (Supplementary Fig. 4). The number of residues that could be equivalenced between any two structures was used to evaluate all possible rooted trees that might represent the evolution of portal proteins. The different rooted trees that could represent the divergence of the portal proteins for the tailed phages A, B, C or D are shown in Fig. 7 and Supplementary Table 7. The evolutionary distance between any pair of these structures can be represented by AB, AC, AD, BC, BD and CD. Two different schemes (*P*−*r*) and (100*P*/*r*) were explored for defining evolutionary distance, where *r* is the number of residues that could be spatially equivalenced between any two structures and *P* is a constant representing the number of residues in the primordial protein. The lengths of the tree branches were represented by the six variables *a*, *b*, *c*, *d*, *e* and *f*. Six linear observational equations can be written for any one tree representing the distance between any pair of structures. For instance, the six observational equations for tree 2 (Fig. 7 and Supplementary Table 7) would be




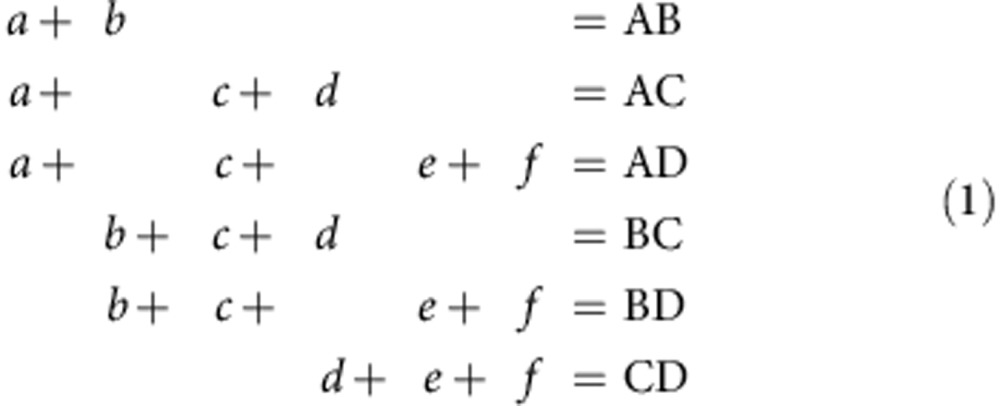




The rate of evolutionary change is likely to be similar for each viral portal protein since its divergence from a common primordial structure. Thus, the distance (*g*) from the tree’s root (*R*) to any one of the current structures (A, B, C or D) should be the same. Hence, four additional observational equations can be written expressing this constraint, resulting in a total of 10 observational equations to determine seven parameters. The additional equations for tree 2 are:




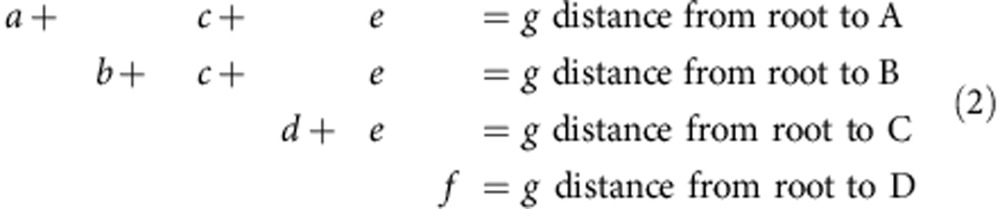




From these 10 observational equations, seven normal equations were calculated and solved for *a*, *b*, *c*, *d*, *e*, *f* and *g*. The resultant values can be substituted into the 10 observational equations to calculate values for AB, AC and so on in equations (1) and (2). An ‘*R*’ factor was then calculated between the observed and calculated distances (Dist) (AB, AC, AD, BC, BD and CD) according to




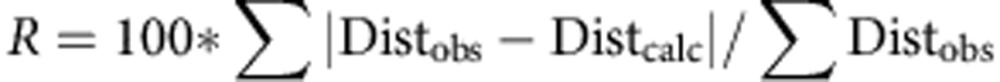




for each of the six possible trees. Two different *R* values were calculated to evaluate the tree preferences. *R*_all_ used both equations (1) and (2), whereas *R*_pair_ included only equation (1). Thus, *R*_pair_ omits the assumption the evolutionary distance of each structure from the root of the tree at *R* is the same.

The lower the *R* factor the more accurate was the tree for representing the observed distances and, hence, presumably the evolutionary events that led to the current portal proteins. Note that using 100*P*/*r* or (*P*−*r*) to measure evolutionary did not substantially change the hierarchy of acceptable trees. Changing the value of *P* made very little difference to the results.

## Additional information

**How to cite this article:** Sun, L. *et al*. Cryo-EM structure of the bacteriophage T4 portal protein assembly at near-atomic resolution. *Nat. Commun.* 6:7548 doi: 10.1038/ncomms8548 (2015).

## Supplementary Material

Supplementary Information Supplementary Figures 1-6 and Supplementary Tables 1-8
