# Supplementary material for: Cryo-EM structure of the bacteriophage T4 portal protein assembly at near-atomic resolution
Source: Nat Commun. 2015 Jul 6;6:7548. doi: 10.1038/ncomms8548 (PMC4493910; doi:10.1038/ncomms8548)
Supplement: Supplementary Information — Supplementary Figures 1-6 and Supplementary Tables 1-8 [file ncomms8548-s1.pdf]

Cryo-EM structure of the bacteriophage T4 portal protein assembly at near-atomic resolution

Lei Sun, Xinzheng Zhang, Song Gao, Prashant A. Rao, Victor Padilla-Sanchez, Zhenguo Chen,

Siyang Sun, Ye Xiang, Sriram Subramaniam, Venigalla B. Rao & Michael G. Rossmann

Supplementary Information consists of:

Supplementary Figures 1 through 6

Supplementary Tables 1 through 8

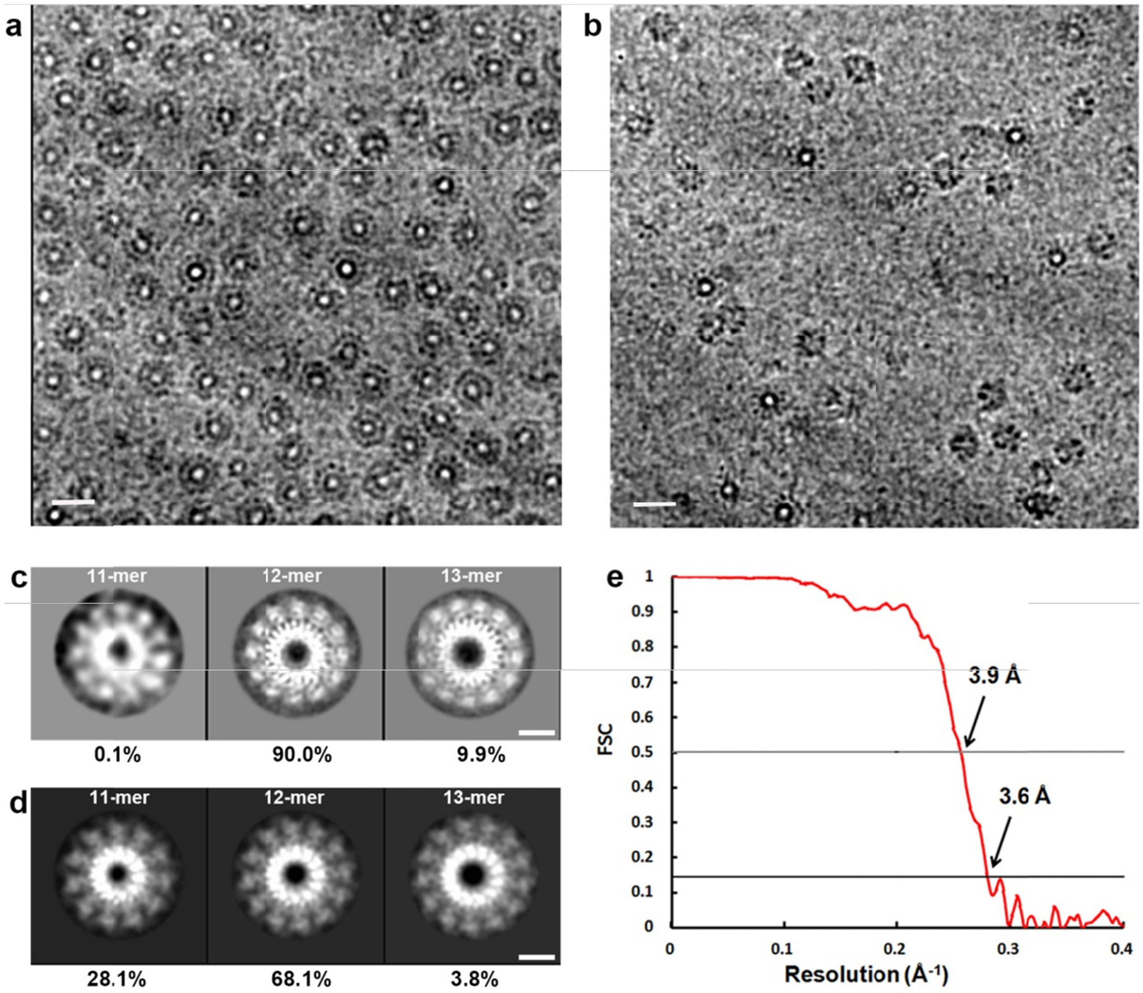

Supplementary Figure 1. (**a-b**) Representative electron micrograph of gp20-N74 embedded in a thin layer of vitreous ice recorded at a defocus of 2μm. Scale bar represents 20nm. (**a**) The particles had a top-view preference. (**b**) When octyl-β-glucoside was added there was an increased percentage of side-views. **(c)** Top views of reference-free 2D class averages of gp20-N74 particles. Scale bar represents 5nm. (**d**) Reference-free 2D class averages of gp20 N74 with the tunnel-loop (residues 377-388) deleted. This mutant produced much more 11-mers and fewer 13-mers as compared to gp20-N74 in which the tunnel loop is present. Scale bar represents 5nm. (**e**) Gold-standard FSC curve for the 3D reconstruction, marked with resolutions corresponding to FSC=0.5 and 0.143.

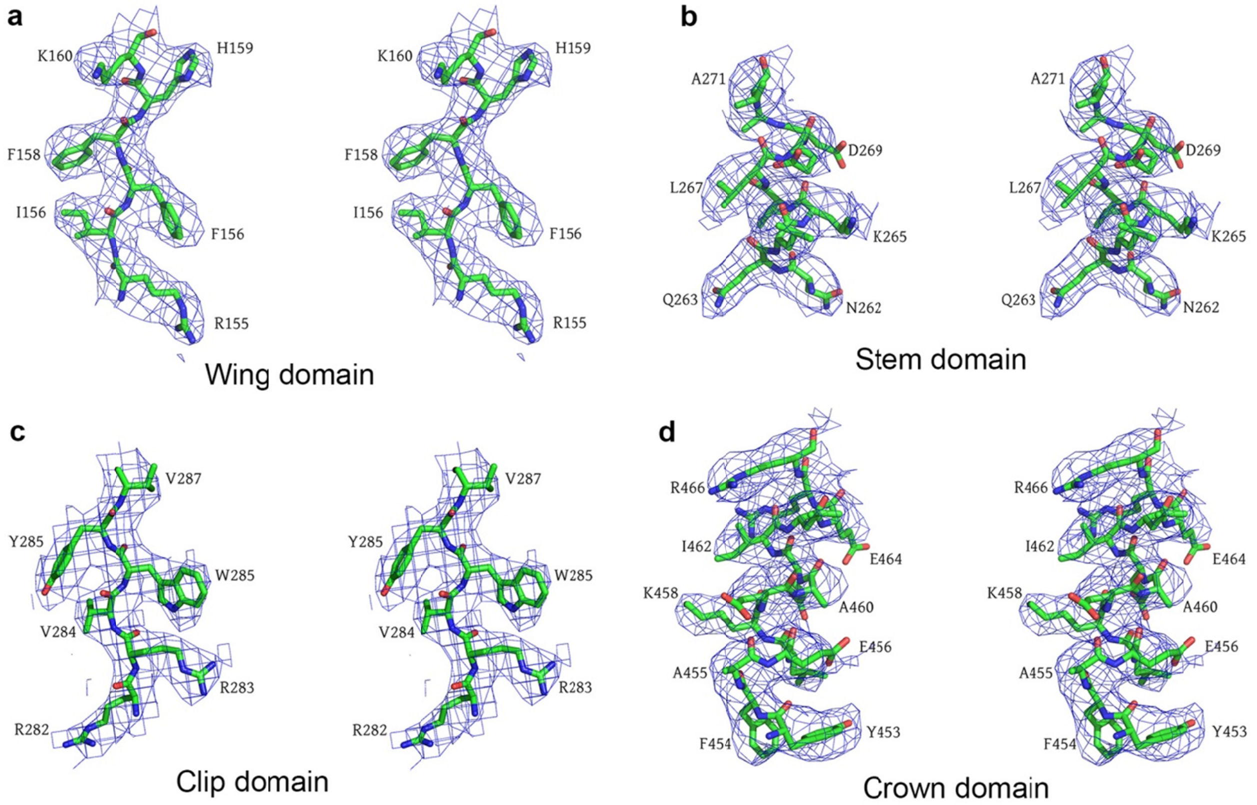

Supplementary Figure 2. Stereographic views of cryo-EM densities, showing selected regions of gp20 at 3.6Å resolution. (**a–d**) Representative cryo-EM densities (blue mesh) are superimposed on the atomic model (main chain in green) for various domains, as indicated.

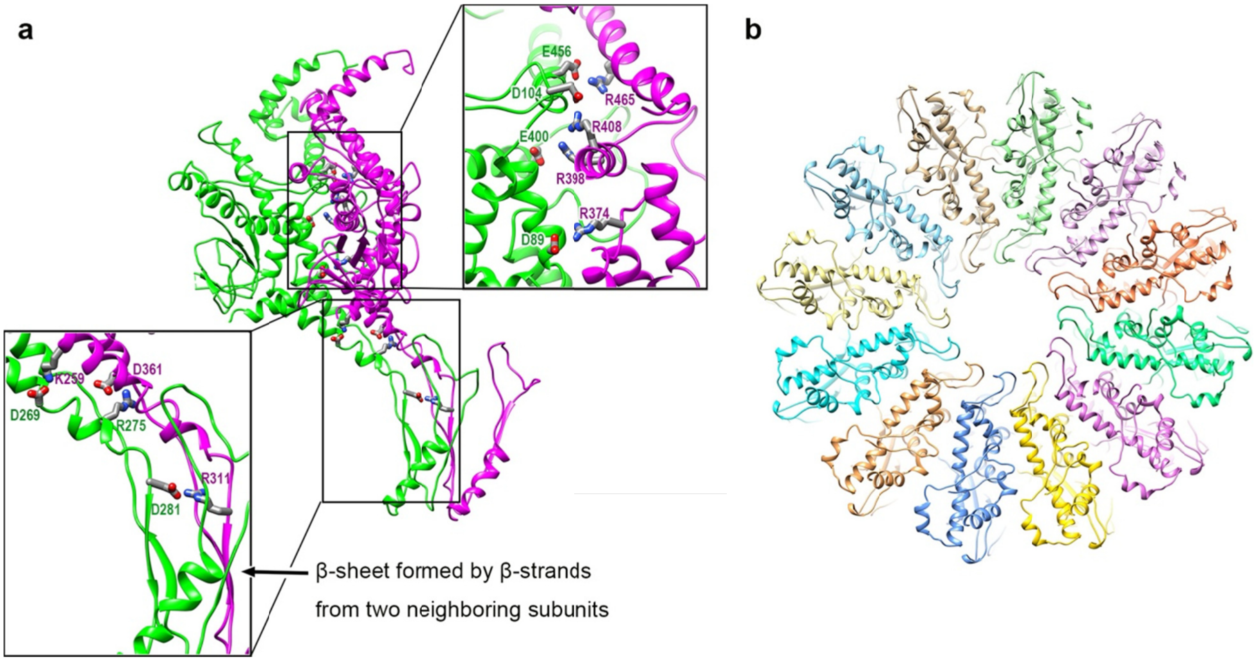

Supplementary Figure 3. Interactions between neighboring subunits. (**a**) The interactions between two neighboring subunits. The residues forming salt-bridges between two subunits (purple and green) are shown as sticks. (**b**) A thin section of the tunnel-loop region of T4 portal assembly, showing how the tunnel-loop interactions between neighboring subunits stabilize the portal dodecamers.

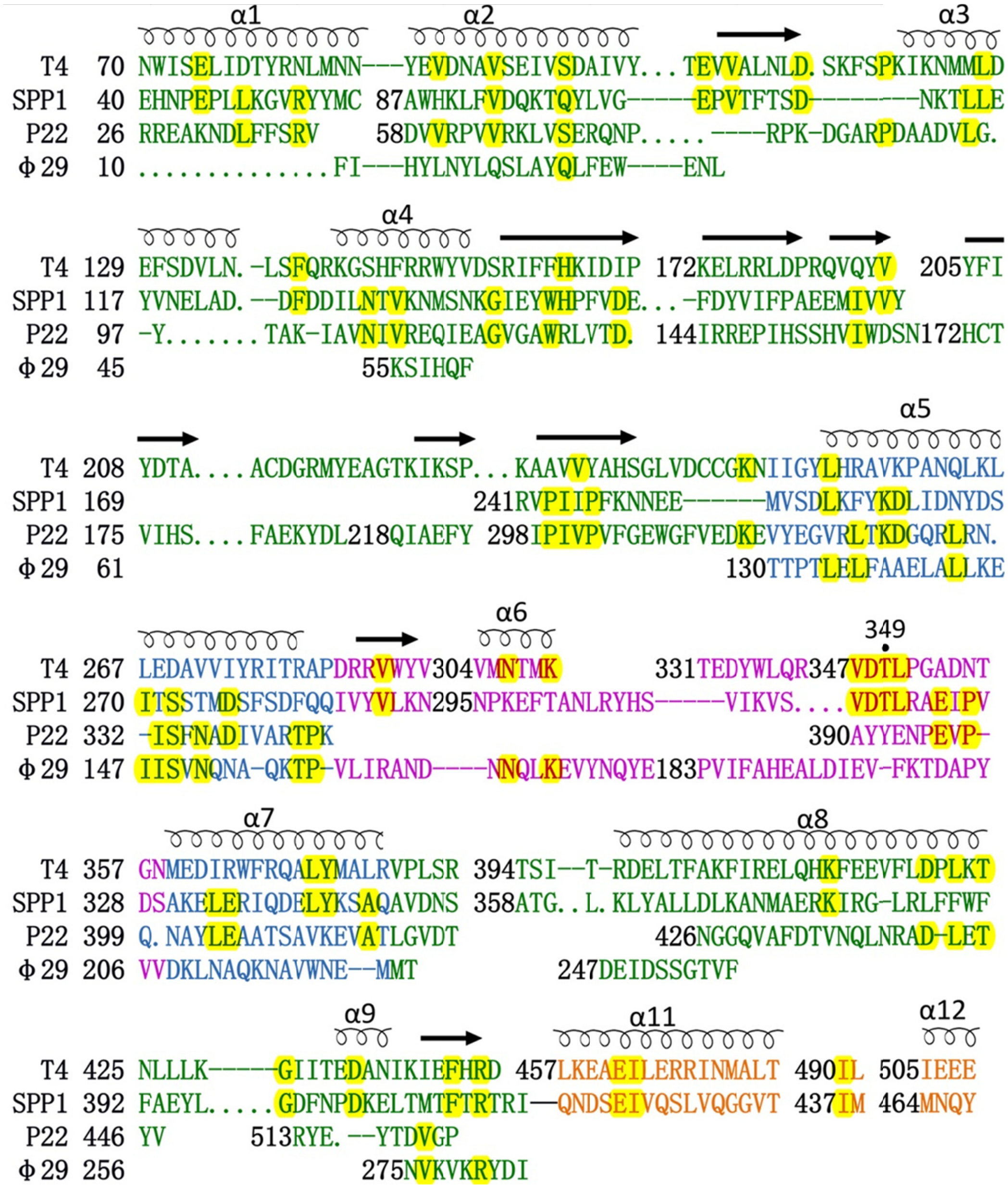

Supplementary Figure 4. Structural alignment of four portal proteins. These alignments are based on pairwise superimposition of whole portal structures. Additional aligned residues would have been found if the alignments had been performed one domain at a time. Dots represent residues that cannot be structurally aligned. Dashes represent gaps relative to the other proteins. Blanks represent larger unalignable insertions. The sequence number is given of the first aligned residue after an unaligned stretch. Highlighted residues are conserved residues in two or more portal proteins. The residues in the wing, stem, clip and crown domains are colored in green, blue, purple and orange, respectively.

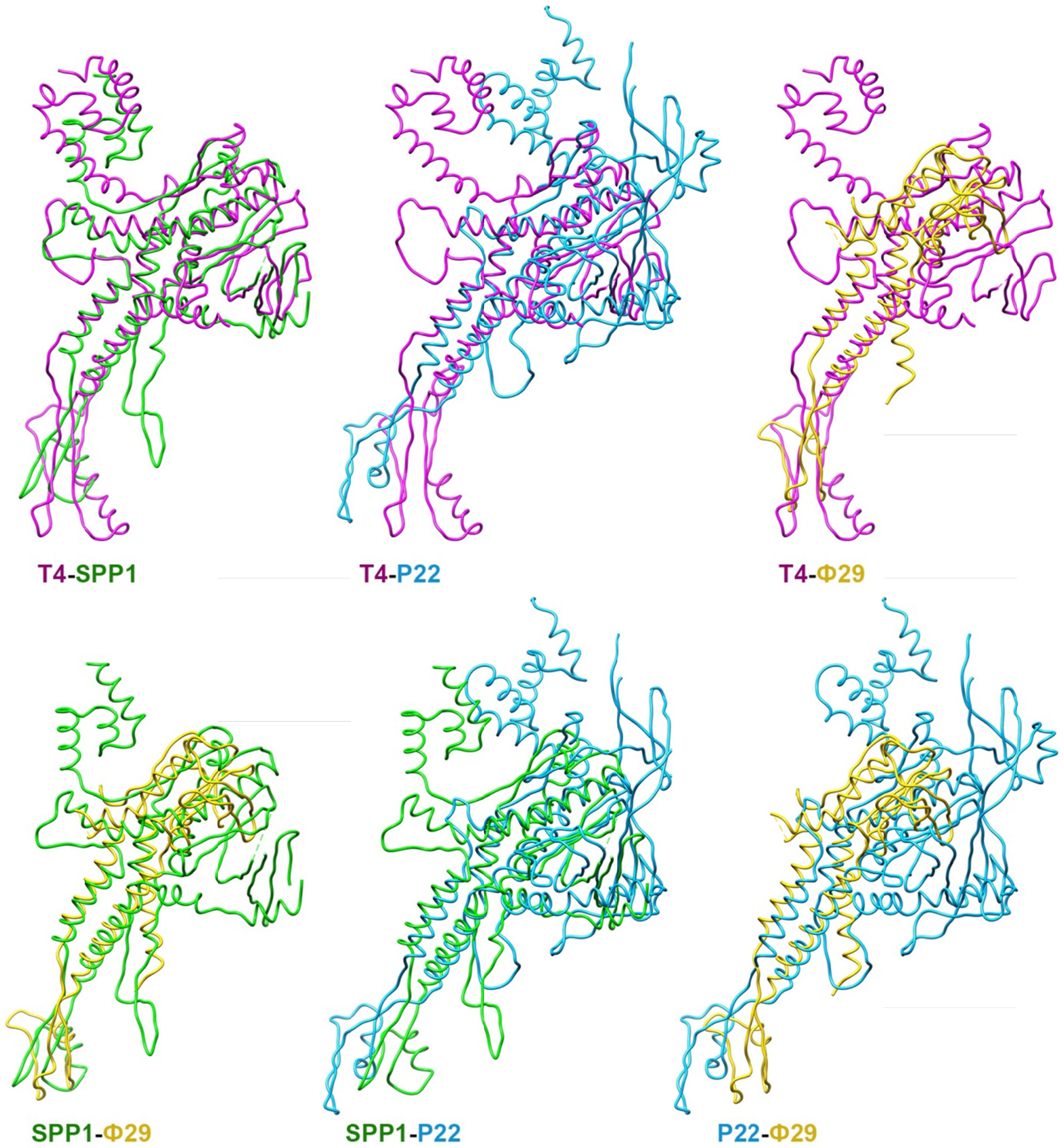

Supplementary Figure 5. Pairwise superimposition of the structures of the four known portal proteins.

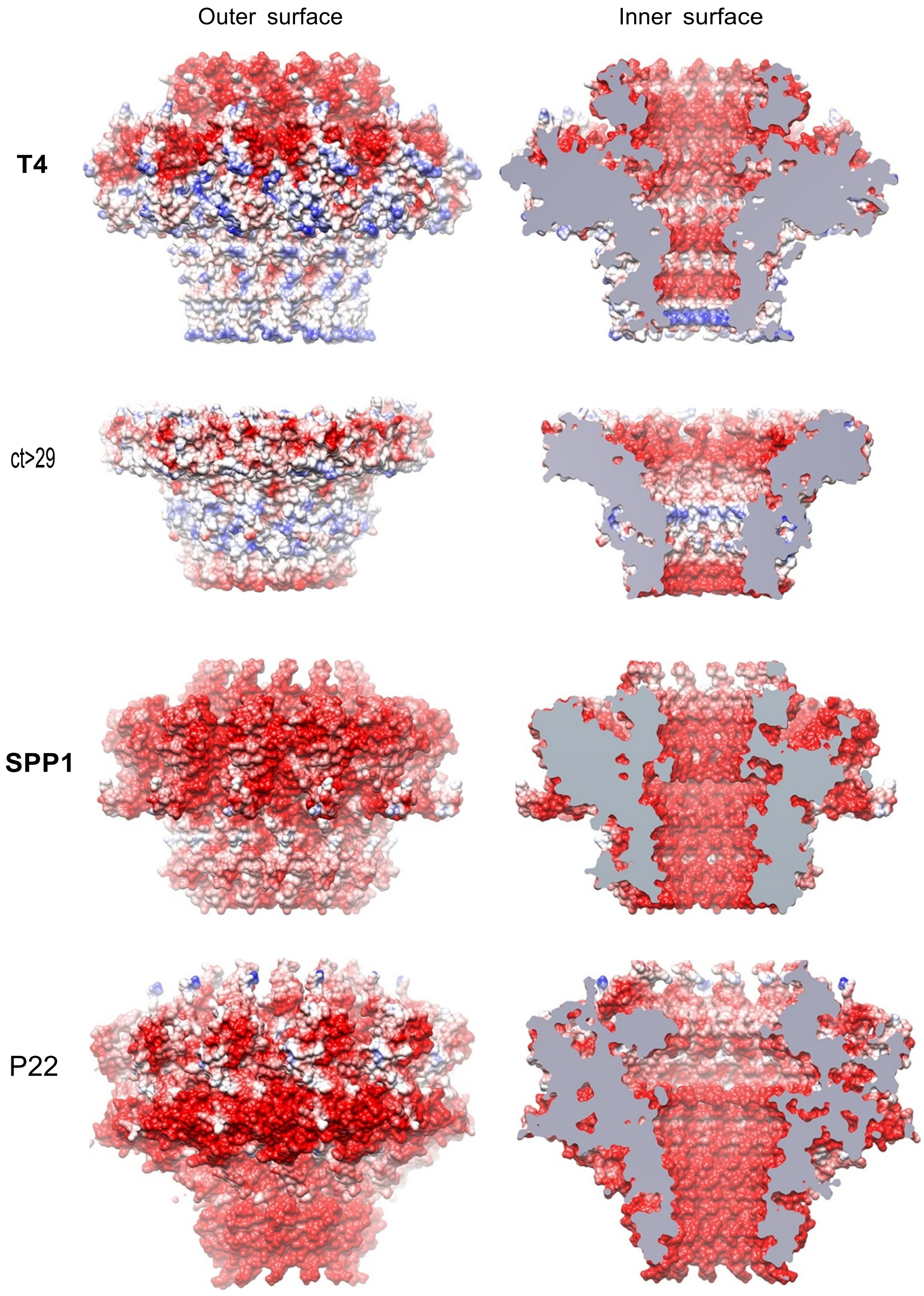

Supplementary Figure 6. Surface charge of four portal proteins whose structures have been determined. *Blue* and *red* colors correspond to positive and negative potential of lOkT e-, respectively.

Supplementary Table 1. gp20 recombinant constructs and their phenotypes.

**gp20 constructs**	**Phenotypes**
WT (aa 1-524) (N-His)	Forms soluble and insoluble aggregates<br>Assembles heads<br>Binds to the ends of linear DNA.
Tunnel loop (aa 377-388) deleted	Assembles heads<br>Packages DNA *in vitro*<br>Packages shorter genomes *in vivo*
aa 1-494 (N-His)	Forms insoluble aggregates
aa 221-503 (N-His)	Forms aggregates and heterodisperse oligomers
aa 52-524 (N-His)	Binds non-specifically to other proteins when overexpressed. Forms aggregates
aa 74-433 (N-His)	No expression
aa 74-450 (N-His)	Forms insoluble aggregates
aa 1-433 (N-His)	No expression
aa 1-478 (N-His)	No expression
aa 1-450 (N-His)	No expression
aa 103-450 (N-His)	Very low expression<br>Forms insoluble aggregates
aa 66-524 (N-His)	Mostly soluble<br>Forms 12-mers
aa 69-524 (N-His)	Mostly soluble<br>Forms 12-mers
aa 72-524 (N-His)	Mostly soluble<br>Forms 12-mers
aa 78-524 (N-His)	Mostly soluble<br>Forms 12-mers
aa 87-524 (N-His)	Forms insoluble aggregates
aa 103-524 (N-His)	Forms insoluble aggregates
aa 119-524	No expression
aa 63-494 (N-His)	Forms aggregates and monomers
aa 63-503 (N-His)	Forms aggregates and monomers
aa 71-494 (N-His)	Forms aggregates
aa 71-503 (N-His)	Forms aggregates
aa 434-524 (N-His)	Forms heterodisperse oligomers
aa 450-524 (N-His)	Forms heterodisperse oligomers
N15 (aa 15-524) (N-His)	Partly soluble<br>Forms 12-mers
**N63 (aa 63-524) (N-His)**	Mostly soluble<br>Forms 12-mers<br>**Cannot assemble heads**
**N74 (aa 74-524) (N-His)**	Mostly soluble<br>Forms 12-mers<br>**Cannot assemble heads**

**gp20 constructs**	**Phenotypes**
N63 Tunnel loop (aa 377-388) deletion	Forms 12-mers and 11-mers<br>Assembles heads<br>Packages DNA *in vitro*<br>Packages shorter genomes *in vivo*
gp13[*] (aa 1-301) (N-His) co-express with gp20 N74	Forms 12-mers
gp20 N74 (N-His) co-express with gp22[**] (aa 1-269)	Forms 12-mers
gp13 (1-301 N-His) co-express with gp20 N15	Forms 12-mers
gp20 N15 N-His co-express with gp22	Very low expression
gp13 (1-301 N-His) co-express gp20 N74 with tunnel loop (377–388) deletion	Forms 12-mers and 11-mers
gp20 N74 tunnel loop (377–388) deletion (N-His) co-express with gp22	Forms 12-mersand 11-mers
N30 aa 30-524	Forms 12-mers
gp13 (1-301 N-His) co-express gp20 N30 no-His	Forms 12-mers
Channel loop (271–284) deletion	Assembles heads<br>Cannot package DNA
T277A	Assembles heads<br>Cannot package DNA
T277A-R278A	Assembles heads<br>Cannot package DNA
N291A-M292A	Assembles heads gp17 binds to head Cannot package DNA
R295A-K296A	Assembles heads gp17 binds to head Cannot package DNA
R338A-R339A	Assembles heads gp17 binds to head Cannot package DNA
D340A-K342A	Assembles heads gp17 binds to head Cannot package DNA

[*]gp13 is part of the neck assembly. It assembles onto the portal as a dodecamer.

[**]gp22 is the major scaffolding protein. It associates with gp20 to initiate the assembly of the major capsid protein (gp23) into heads. This construct is used for cryo-EM data collection

Supplementary Table 2. Refinement of the gp20 structure.

	gp20-N74
Resolution (Å)	3.6
Space group	P1
Cell dimension a, b, c (Å)	272, 272, 272
Cell angle α=β=γ (°)	90, 90, 90
No. of Reflections	880491
R_{work} / R_{free}*	0.26/0.27
No. residues	70-516
R.m.s. deviations	
Bond lengths (Å)	0.01
Bond angles (°)	1.4
Ramachandran plot values	
Most favored (%)	87.3
Generously allowed (%)	10.8
Disallowed regions (%)	1.9

*R_{free} was calculated for 5% of reflections randomly excluded from the refinement

Supplementary Table 3. Interface between T4 portal protein gp20 and the major capsid protein gp23.

gp20	gp23
MYEAGTK(221-227), ITE(189-191), K311,	TKSVF (409-413) GPNEM (440-443) Q273, Q276, K279

Supplementary Table 4. Portal protein domain boundaries.

Domain	T4	SPP1	P22	ϕ29
Wing	70-254	29-253	5-317	11-129
Stem	255-278	254-283	318-344	130-156
Clip	279-358	284-324	345-396	157-204
Stem	359-377	325-347	397-420	205-228
Wing	378-451	348-420	421-526	229-285
Crown	452-524	421-467	527-602	
Helical barrel			603-725	

Supplementary Table 5. Structurally equivalenced residues between any pair of portal subunits.*

	T4	SPP1	P22	ϕ29
T4	428	10	6	5
SPP1	248(239)	400	11	7
P22	217(208)	179(203)	569	12
ϕ29	129(123)	121(113)	82(89)	271

*The bottom left (in green) shows the number of aligned residues as found by the HOMOLOGY program (in parenthesis) and the numbers of aligned residues after manual adjustments. The top right (in blue) shows the percentages of completely conserved residues based on the manually adjusted alignment.

Supplementary Table 6. Percentage of structurally equivalence residues between pairs of portals, based on the manually adjusted HOMOLOGY alignment.*

	T4	SPP1	P22	ϕ29
T4 (524)	100	47	41	24
SPP1 (503)	49	100	36	24
P22 (603)	36	30	100	14
ϕ29 (309)	42	39	27	100

*The percentage is calculated with respect to the number of residues in each portal protein given in parentheses for each row.

Supplementary Table 7. Trees and branches for all possible trees that might represent the divergent evolution of four known portal proteins.*

Tree1													
Distance	A	B	C	D	a	b	c	d	e	f	g	R_{all}	R_{pair}
P-r	T4	SPP1	P22	ϕ29	53	58	39	-36	101	160	94	10.8	5.2
100P/r	T4	SPP1	P22	ϕ29	68	78	44	-80	154	239	117	12.1	5.7
P-r	T4	P22	SPP1	ϕ29	59	83	22	-25	80	157	93	15.7	6.8
100P/r	T4	P22	SPP1	ϕ29	62	107	40	-30	95	215	125	19.5	8.4
P-r	SPP1	P22	T4	ϕ29	64	83	20	-20	73	154	94	16.1	7.3
100P/r	SPP1	P22	T4	ϕ29	69	104	41	-15	78	207	127	19.9	8.8
Tree2													
Distance	A	B	C	D	a	b	c	d	e	f	g	R_{all}	R_{pair}
**P-r**	**T4**	**SPP1**	**P22**	**ϕ29**	**53**	**58**	**9**	**80**	**51**	**121**	**121**	**3.4**	**1.7**
100P/r	T4	SPP1	P22	ϕ29	68	78	-12	109	87	165	165	9.1	4.7
P-r	T4	P22	SPP1	ϕ29	59	83	-5	63	56	121	121	4.6	2.3
100P/r	T4	P22	SPP1	ϕ29	62	107	3	72	82	165	165	10	6.3
P-r	SPP1	P22	T4	ϕ29	64	83	-5	58	56	121	121	4.7	2.4
100P/r	SPP1	P22	T4	ϕ29	69	104	9	62	80	165	165	9.6	5.7
P-r	SPP1	P22	ϕ29	T4	64	83	26	150	-36	80	80	12.4	6.5
100P/r	SPP1	P22	ϕ29	T4	69	104	58	208	-65	100	100	14.8	9
P-r	SPP1	ϕ29	P22	T4	80	157	-10	95	-24	80	80	15.2	7.6
100P/r	SPP1	ϕ29	P22	T4	95	215	-2	130	-45	100	100	18.4	9.2
P-r	P22	ϕ29	SPP1	T4	101	160	-16	78	-22	80	80	14.7	7.4
100P/r	P22	ϕ29	SPP1	T4	154	239	-56	101	-27	100	100	15.1	7.5
P-r	T4	ϕ29	P22	SPP1	73	154	-6	94	-21	83	83	16.1	8.1
100P/r	T4	ϕ29	P22	SPP1	78	207	12	126	-40	105	105	20.1	10
P-r	T4	P22	ϕ29	SPP1	59	83	24	149	-31	83	83	13.7	7.3
100P/r	T4	P22	ϕ29	SPP1	62	107	48	207	-52	105	105	17.9	11.1
P-r	ϕ29	P22	T4	SPP1	160	101	-18	72	-17	83	83	15.2	7.6
100P/r	ϕ29	P22	T4	SPP1	239	154	-60	90	-16	105	105	15.7	7.9
P-r	SPP1	ϕ29	T4	P22	80	157	-19	69	2	92	92	17.3	8.7
100P/r	SPP1	ϕ29	T4	P22	95	215	-18	79	5	122	122	21.4	10.7
P-r	T4	ϕ29	SPP1	P22	73	154	-13	73	0	92	92	17.7	8.9
100P/r	T4	ϕ29	SPP1	P22	78	207	-1	87	-1	122	122	22.5	11.2
P-r	SPP1	T4	ϕ29	P22	58	53	31	145	-15	92	92	14.3	7.3
100P/r	SPP1	T4	ϕ29	P22	78	68	20	204	-8	122	122	21.2	11.1

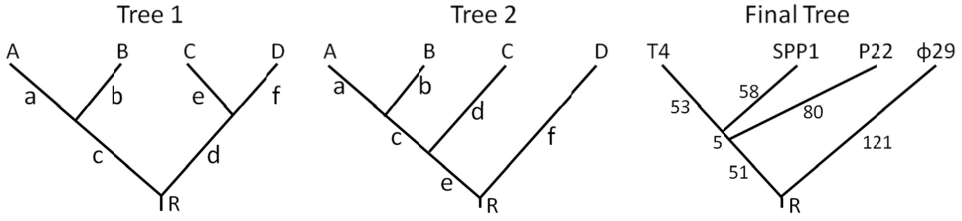

*A, B, C, D determines the nature of the tree as shown in the bottom panel. a, b, c, d, e, f and g gives the branch lengths in each tree. R_{all} and R_{pair} are residual measures of the tree quality. P-r and 100/**r** are alternative ways of estimating the evolutionary distance between pairs of structures where r is the number of the structural aligned residues, and P is taken as 350, although the result is not sensitive to its actual value.

Supplementary Table 8. Difference in angles (measured in degrees) between equivalent domains.*

	Wing	Stem	Clip	Crown
T4 vs. SPP1	8	6	36	32
T4 vs. P22	6	15	26	60
T4 vs. ϕ29	35	6	9	-
SPP1 vs. P22	14	30	39	60
SPP1 vs. ϕ29	17	5	36	-
P22 vs. ϕ29	29	7	41	-

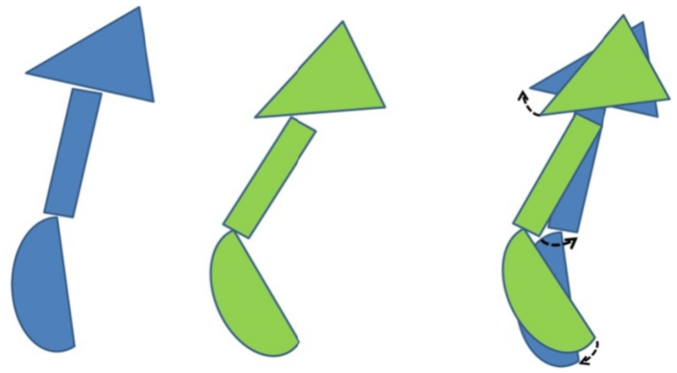

*The best average superposition (right) was determined for any two portals (blue and green). Then the difference in domain orientation (black arrows) was determined between each pair of equivalent domains.
